# Enzymatic synthesis of new antimicrobial peptides for food purposes

**DOI:** 10.3389/fmicb.2023.1153135

**Published:** 2023-05-16

**Authors:** Mauricio Adaro, Ángel Gabriel Salinas Ibáñez, Anabella Lucia Origone, Diego Vallés, Fanny Guzmán, Alba Vega, Sonia Barberis

**Affiliations:** ^1^Laboratorio de Bromatología, Facultad de Química, Bioquímica y Farmacia, Universidad Nacional de San Luis, San Luis, Argentina; ^2^Instituto de Física Aplicada (INFAP) – CCT - San Luis - CONICET, Piso, San Luis, Argentina; ^3^Laboratorio de Microbiología e Inmunología, Facultad de Química, Bioquímica y Farmacia, Universidad Nacional de San Luis, San Luis, Argentina; ^4^Laboratorio de Biocatalizadores y sus Aplicaciones, Instituto de Química Biológica, Facultad de Ciencias, Universidad de la República (UdelaR), Montevideo, Uruguay; ^5^Laboratorio de Péptidos, Núcleo de Biotecnología Curauma, Pontificia Universidad Católica de Valparaíso, Curauma, Valparaíso, Chile

**Keywords:** antibacterial peptide, enzymatic synthesis of peptides, Ile-Gln (IQ), novel peptide against sensitive and SDR Gram positive and Gram negative strains, food preservation

## Abstract

Growing consumer awareness of the potential negative health effects of synthetic antibiotics has prompted the search for more natural preservatives that can improve the safety and quality of food. In this study we report the enzymatic synthesis of N-α-[Carbobenzyloxy]-Ile-Gln (Z-IQ) which is the precursor of Ile-Gln (IQ), a new antibacterial dipeptide, using an aqueous–organic biphasic system formed by 50% (v/v) ethyl acetate in 0.1 M Tris – HCl buffer pH 8. A partially purified proteolytic extract from the fruits of *Solanum granuloso leprosum,* named granulosain, proved to be a robust biocatalyst for the synthesis of Z-IQ, eliciting 71 ± 0.10% maximal peptide yield in the above described conditions. After cleaving and purifying IQ dipeptide, antimicrobial activity was assayed against *Staphylococcus aureus* ATCC 25923, *Staphylococcus hominis* A17771, and *Staphylococcus aureus* C00195, and MIC values between 118 ± 0.01 μg/mL and 133.7 ± 0.05 μg/mL were obtained. In addition, IQ showed MIC of 82.4 ± 0.01 μg/mL and 85.0 ± 0.00 μg/mL against *Escherichia coli* ATCC 25922 and *Escherichia coli* A17683, respectively. IQ did not show inhibitory activity against single-drug resistance (SDR) strains, such as *Klebsiella oxytoca* A19438 (SDR) and *Pseudomonas aeruginosa* C00213 (SDR), and against multidrug-resistant *Enterococcus faecalis* I00125 (MDR). IQ also caused growth inhibition of *Helicobacter pylori* NCTC 11638 and three wild-type *H. pylori* strains, which are sensitive to AML, MTZ, LEV and CLA (*H. pylori* 659), resistant to LEV (*H. pylori* 661 SDR), and resistant to MTZ (*H. pylori* 662 SDR). Finally, this study contributes with a new dipeptide (IQ) that can be used as an antimicrobial agent for food preservation or as a safe ingredient of functional foods.

## Introduction

The International Network of Food Safety Authorities (INFOSAN), the World Health Organization (WHO) and the Food and Agriculture Organization of the United Nations (FAO), estimate that 600 million people in the world (almost 1 out of 10) suffer from diseases from eating contaminated food and 420,000 die each year. Children under 5 years of age make up 40% of foodborne illness, with 125,000 deaths each year (CDC – Centers for Disease Control and Prevention, 2018). In the food industry, the control of pathogenic bacteria is achieved through different preservation methods (thermal treatment, salting, acidification, drying) and the addition of synthetic antibiotics in those foods that are authorized by the food regulations of each country (Sabillón et al., 2016).

Synthetic antibiotics are chemical compounds that are added to food, packaging, food contact surfaces, or food processing environments to inhibit microbial growth or kill microorganisms. The main functions of antimicrobials in food are: (1) control natural spoilage processes (food preservation) and (2) prevent/control the growth of food spoilage microorganisms and/or pathogens (food safety) (Davidson et al., 2014). However, the use of synthetic antibiotics can cause undesirable effects in the consumer; the development of bacterial resistance to antibiotics being a true global public health emergency (Hashempour-Baltork et al., 2019).

Growing consumer awareness of the potential negative health impact of synthetic antibiotics has prompted the search for alternative, more natural preservatives that can improve food safety and quality.

According to WHO, *Helicobacter pylori* is 1 of the 16 microorganisms causing the greatest threats to human health with a high impact on public health (World Health Organization, 2022).

The most frequent *H. pylori* pathologies are related to chronic gastritis and gastric ulcer, affecting more than half the world’s population. In addition, infection with *H. pylori* is the strongest recognized risk factor for gastric adenocarcinoma and lymphoma (Dang and Graham, 2017). *H. pylori* infections are usually treated with at least two different antibiotics at once, such as clarithromycin (CLA), metronidazole (MTZ), levofloxacin (LEV) and amoxicillin (AML) (Marcus et al., 2016). Treatment also include other medications, such as proton pump inhibitor and bismuth subsalicylate. However, the success of those treatments is decreasing due to bacterial resistance to antibiotics (Malfertheiner et al., 2017; Ozturk et al., 2017).

There is a little history of the use of antimicrobial peptides (AMPs) against *H. pylori* (Xiong et al., 2017; Guzman et al., 2018), but it was reported that stomach cells express AMP during infection and could play a role in the immune response (Gopal et al., 2014). This suggests that the use of AMPs in functional foods could be an effective strategy for the prevention of *H. pylori* infection.

The IQ-motif occurs in a wide range of eukaryotic proteins and peptides, and some of them were associated with antimicrobial activity (McLean et al., 2013). The literature reports that 3IQ1 and 3IQ2 have displayed MIC values toward *E. coli* and *S. aureus* which are similar to or better than those seen with the antimicrobial peptide cathelicidin (LL-37; McLean et al., 2013; Neshani et al., 2018; Kang et al., 2019). Instead, other IQ-motif peptides such as 2IQ4 and the anionic peptide 2IQ1 displayed no bioactivity (McLean et al., 2013). To the best of our knowledge, there are no literature reports on the enzymatic synthesis of IQ dipeptide and its potential antimicrobial activity.

Peptidase-catalyzed synthesis of peptide bonds has shown several advantages over other methodologies used to produce peptides (Wall et al., 1997; Barberis et al., 2008; Wakasa et al., 2011; Agyei and Danquah, 2012; Ortíz-Martínez et al., 2014; Fosgerau and Hoffmann, 2015; Hartsough et al., 2015; Limón et al., 2015). Enzymes are exquisitely selective catalysts, capable of choosing a single substrate from a large number of similar compounds and reacting with high specificity under mild conditions. In addition, enzymatic processes are usually cheaper and more sustainable than chemical processes (Barberis et al., 2018). However, only some plant proteases have been used in peptide synthesis processes (Origone et al., 2018, 2020; Adaro et al., 2021).

Vallés et al. (2004) reported the isolation, partial purification, and characterization of a proteolytic extract obtained by grinding the ripe fruits of *Solanum granuloso leprosum* (Solanaceae), a South American native plant. The crude extract had maximum activity in the pH range from 5.2 to 7.6 and temperatures between 50°C and 55°C. It showed a remarkable stability at pH 7.6 and freezing temperature (−18°C). The main purified fraction (granulosain I) displayed optimal proteolytic activity in the pH range from 7 to 8.6 and high stability at ≤4°C. The crude enzyme extract and the purified fraction were tested against several protease inhibitors, and the results showed that they belong to the cysteine protease type (Vallés et al., 2004).

In industrial processes, minimum enzyme purity criteria are often applied for economic reasons (Abreu and de Figueiredo, 2019). Consequently, a partially purified extract was used as biocatalyst in this study.

Synthesis of peptides will not proceed efficiently in aqueous medium where the hydrolytic potential of the enzyme prevails. The design of reaction media is a major challenge for peptide synthesis, since proteases, different from lipases, are not structurally conditioned to act in such environments (Illanes et al., 2009; Origone et al., 2020).

The aim of this work was to study the ability of soluble granulosain (the partially purified proteolytic extract from the fruits of *Solanum granuloso leprosum*) as a biocatalyst for the synthesis of N-α-[Carbobenzyloxy]-Ile-Gln (Z-IQ), which is a precursor of IQ, a novel dipeptide potentially useful as antibacterial agent for food applications. Synthesis was carried out under kinetic and thermodynamic control; in 50% (v/v) ethyl acetate in 0.1 M Tris – HCl buffer pH 8. The results were compared with the amine-terminal IQ analogous peptide obtained by chemical synthesis.

## Materials and methods

### Reagents

(N-α-[(benzyloxy)carbonyl]-amino acid-p-nitrophenyl ester hydrochloride (≥ 98.0%), N-α-[(benzyloxy)carbonyl]-L-Ile (Z-Ile-OH) (≥ 98.0%), N-α-benzoyl-DL-arginine-p-nitroanilide (BApNA, ≥ 96.0%), monobasic sodium orthophosphate anhydrous (≥ 99.0%), Tris–HCl (Tris(hydroxymethyl) aminomethane hydrochloride, ≥ 99.0%), 4-nitrophenol (≥ 99.0%), 4-nitroaniline (≥ 99.0%), 2-mercaptoethanol (≥ 99.0%), trifluoroacetic acid for HPLC (≥ 99.0%), and bovine serum albumin by agarose gel electrophoresis (BSA, ≥ 99.0%) were bought from Sigma-Aldrich (St. Louis, MO, United States).

L-cysteine hydrochloride monohydrate (≥ 98%), NaOH (≥ 98.0%), phosphate buffer solution (Certipur®, pH 7.00, at 20°C), bicarbonate buffer solution pH 10 (100 mM sodium carbonate and 100 mM sodium bicarbonate), sulfuric acid (98%) and NaOH (≥ 98.0%), were bought from Merck KGaA (Darmstadt, Germany). Acetonitrile, acetone, 1,2-dichloroethane, isopropyl ether, n-hexane, toluene, 2-chlorotoluene, 1-heptanol, phenyl acetone and ethyl acetate, piperidine, dichloromethane (DCM), N,N-dimethylformamide (DMF) (gradient grade for liquid chromatography LiChrosolv®), methanol (gradient grade for liquid chromatography LiChrosolv®), formic acid (98–100% for HPLC LiChropur™) and 0.5 nm molecular sieve beads, were also purchased from Merck KGaA (Darmstadt, Germany). Ethylenedinitrilotetraacetic acid (EDTA, disodium salt, dehydrate, molecular biology grade) was purchased from Calbiochem (San Diego, CA, USA).

### Preparation of partially purified enzymatic extract

*Solanum granuloso leprosum* (Solanaceae), popularly known as fumo bravo, is a pioneer species of the Missionary Forest in Argentina, but it also inhabits Brazil and Uruguay (Burkart, 1979). The orange globular fruits of *Solanum granuloso-leprosum* were harvested in an experimental plantation of Montevideo, Uruguay.

The partially purified proteolytic extract from the fruits of *Solanum granuloso leprosum,* was obtained according to Vallés et al. (2004) and it was lyophilized for later use as biocatalyst. The ripe fruits were ground together with abrasives and centrifuged at 6,654 g for 30 min at 4°C (Vallés et al., 2004). The supernatant was filtered through gauze and the filtrate was called crude extract. A portion (8 mL) of the crude extract was mixed with an equal volume of cold ethanol (−20°C) under gentle stirring, left to settle for 10 min at −20°C, and then centrifuged at 16,000 g for 20 min at 4°C (Scopes, 1994). The precipitate was suspended in 8 mL of 0.2 M phosphate buffer pH 7.0. This partially purified extract containing the soluble proteases, collectively named *granulosain*, was frozen at −20°C for further studies.

### Protein content and enzyme activity assays

Protein concentration in the enzyme extracts was determined by the Bradford protein assay, using BSA as standard (Bradford, 1976). The proteolytic activity of granulosain was determined as the initial rate of hydrolysis against N-α-benzoyl-DL-arginine-p-nitroanilide (BApNA) as substrate. The reaction mixture was prepared by mixing 0.5 mL of partially purified granulosain (2.47 mg protein/mL) with 0.5 mL of 5 mM BApNA containing 20 mM cysteine in 0.1 M Tris–HCl buffer pH 8. After 5 min of incubation at 37°C and 200 rpm, the absorbance of the p-nitroaniline released was measured at 410 nm. Enzymatic units of activity (IU) were obtained by performing a standard curve of p-nitroaniline in 0.1 M Tris–HCl buffer pH 8. An international unit of enzyme activity (IU) is the amount of enzyme that catalyzes the conversion of 1 μmol of substrate per min, under defined operating conditions. A control without substrate under similar conditions was carried out.

### Stability assay method

Stability of soluble granulosain was evaluated at 40°C under nonreactive conditions in 0.1 M Tris–HCl buffer solution pH 8, and in 30, 50 and 70% (v/v) of immiscible organic solvents in 0.1 M Tris–HCl buffer solution pH 8. Immiscible organic solvents were selected from an optimized statistical design of 72 organic solvents based on the principal components analysis, which allowed to cluster the organic solvents according to their physicochemical properties (Barberis et al., 2006).

The residual proteolytic activity of granulosain after 8 h was determined, which was previously evaluated as the end process time for catalyst replenishment. These results allowed to select the most promising liquid – liquid aqueous – organic media for the peptide synthesis reaction.

### Amino acid preference assay

The amino acid preference assay of granulosain was performed against 12 N-α-[Carbobenzyloxy]-amino-p-nitrophenyl esters as substrates, according to the protocol described by Priolo et al. (2001). The enzyme activity unit (Ucbz) was established as the amount of enzyme that cleaves 1.0 μmol of p-nitrophenol per min under the reaction conditions. The most preferred substrate was selected as an acyl donor for peptide synthesis.

### Enzymatic synthesis of Z-IQ peptide

Based on the granulosain preferences for synthetic amino acid derivatives, N-α-[(benzyloxy)carbonyl]-L-Isoleucine-p-nitrophenyl ester (Z-Ile-ONp) and L-Glutamine (Gln-OH) were selected as acyl donor and nucleophile, respectively.

The reaction of synthesis under kinetic control (Guzmán et al., 2007) was carried out at 40°C in a system formed by 50% (v/v) of ethyl acetate in 0.1 M Tris–HCl buffer solution pH 8, containing soluble granulosain (2.47 mg/mL, 32.49 ± 0.186 IU/mg), 20 mM 2-mercaptoethanol, 0.392 mM of both Z-Ile-ONp and triethyl ammonium (TEA), and 3.180 mM of Gln-OH. In addition, the synthesis reaction under thermodynamic control (Guzmán et al., 2007) was also carried out, using 0.392 mM N-α-[(benzyloxy)carbonyl]-L-Isoleucine (Z-Ile-OH) as acyl donor (instead of Z-Ile-ONp) under the same reaction conditions.

The synthesis was carried out in a GFL Shaking Incubator Orbital Motion (Model 3,031, Germany) at 200 rpm and several aliquots (1 mL) were withdrawn during 24 h. The reaction was stopped with 0.2 mL of 0.1% (v/v) trifluoroacetic acid (TFA). A control group consisting of the enzyme alone, each individual substrate and all the reagents together but without the enzyme was also carried out.

Substrate, product and byproducts were analyzed by RP-HPLC and identified by mass spectrometry (MS). The cleavage of the Z group to deprotect the α-amino-group was performed using the protocol described by Isidro-Llobet et al. (2009).

The product yield (η) and the degree of conversion (αs) of acyl donor substrate into product were evaluated by equations (1, 2) (Origone et al., 2018).


(1) $$a_{s}=\frac{\left[S_{o}\right]-\left[S_{t}\right]}{\left[S_{o}\right]}100$$

(2) $$\eta =\frac{\left[p\right]}{\left[S_{o}\right]}100$$

Where: [*P*] is the molar concentration of the product after a certain period of time, [*S_o_*] is the initial molar concentration of the acyl donor, [*S_t_*] is the molar concentration of the acyl donor after a certain period of time.

### Chemical synthesis of amine-terminal IQ peptide

For the solid-phase chemical synthesis of the amine-terminal IQ peptide, 0.2 g of 2-chloro-trityl resin (1.6 mmol/g, 100–200 mesh) and 0.1 g of rink amide AM resin (0.59 mmol/g, 100–200 mesh) were used. Resin loading was calculated according to Wanka et al. (2007). The chemical synthesis protocol used in this work was described in previous publications of our research team (Origone et al., 2018). The coupling reaction was validated with the Kaiser test (Sarin et al., 1981). A solution consisting of TFA, H_2_O, and triisopropylsilane (95:2.5:2.5) (v/v) was used to deprotect the amino acid side chains and cleave the peptide from the resin, during 90 min at 200 rpm and at room temperature.

The amine-terminal IQ peptide was precipitated with cooled ethyl ether at −70°C, purified in a C_18_ cartridge (Merck) and lyophilized. Then, its purity and molecular mass was determined by RP-HPLC (Jasco, AS-2055, PW de Meern, Nederland), Electrospray ionization-mass spectrometry (ESI-MS) (LC–MS 2020, Shimadzu, Montevideo, Uruguay) and MALDI-TOF Microflex (Bruker Daltonics, Bremen, Germany).

### Analytical assays of peptide synthesis reactions

#### RP-HPLC

In order to study the reaction kinetics of the enzymatic synthesis of Z-IQ in the liquid - liquid aqueous - organic media which was previously selected, the reaction progress was followed by RP-HPLC (Thermo Electron North America LLC, Palm Beach, FL, United States) using a C_18_ column, 4.60 mm × 250 mm, particle size: 5 μm (Hypersil BSD, Base Silica Deactivated, USA) and UV Detector at λ: 254 nm and 25°C. The injection volume was 20 μL, the flow rate of the mobile phase (50% (v/v) acetonitrile in 0.1 M Tris–HCl buffer pH 3) was 0.8 mL/min.

Enzymatically and chemically synthesized peptides (Z-IQ and IQ) were purified by means of a C_18_ cartridge (Merck) (> 95% purity) and dried in a concentrating device (Thermo Scientific Savant™ SPD131DDA SpeedVac, Madrid, España).

The purified Z-IQ and IQ peptides were analyzed by HPLC (Jasco, AS-2055, PW de Meern, Nederland) with a Photo Diode Array Detector, using a C_18_ column (100 × 4.6 mm, 3.5 μm) (XBridge™ BEH, Waters). Injection volume was 20 μL and mobile phase flowrate was 1 mL/min. The mobile phase consisted of a solution A (2.5% TFA in Mili Q water) and a 30–100% gradient of solution B (2.5% TFA in acetonitrile), during 20 min.

#### Mass spectrometry

Two micro gram of each peptide were analyzed in an electrospray ionization-mass spectrometry (ESI-MS) (LC–MS 2020, Shimadzu, Montevideo, Uruguay) under positive ion mode during 20 min at 350°C and 4.5 kV. Data were evaluated with a LabSolutions software (version 5.42, Shimadzu).

#### MALDI-TOF

One micro liter of each peptide (1 μg/μL) and 1 μL of the CHCA matrix (10 μg/μL α-cyano-4-hydroxicinnamic acid in a solution consisting in 0.1% methanoic acid and 50% acetonitrile) were placed in a micro scout plate. Samples were air dried and analyzed on a MALDI-TOF Microflex (Bruker Daltonics, Bremen, Germany). The equipment operated under reflection detection in positive ion mode and was calibrated with an external standard (700–1800 Da). Spectra were recorded using flexControl software (version 3.0, Bruker Daltonics GmbH).

### Bacterial strains

*S. aureus* ATCC 25923, *E. coli* ATCC 25922, *H. pylori* NCTC 11638 and nine wild-type strains were used in this study. Six Gram positive and Gram negative wild-type strains were isolated from patients in the Microbiology Laboratory of the Regional Polyclinic of San Luis, Argentina. *H. pylori* NCTC 11638 was kindly provided by Dra. Teresa Alarcón Cavero, Microbiology Service of Hospital Universitario de la Princesa, Madrid, Spain. Three wild-type *H. pylori* strains were isolated in our laboratories from biopsy samples of the gastric antrum of patients from San Luis (Argentina). The patients who attended at the healthcare center fulfilled the informed consent form prior to sample collection. *H. pylori* isolates were identified by microscopy, urease, catalase, and oxidase tests. All strains were stored at - 80°C in trypticase soy broth (TSB, Britania) with 20% glycerol (Biopack, Buenos Aires, Argentina). Antimicrobial susceptibility was tested according to the Clinical and Laboratory Standards Institute (Clinical and Laboratory Standards Institute (CLSI), 2015).

### Determination of antibacterial activity

The antibacterial activity of IQ-OH and IQ-NH_2_ against 12 Gram positive and Gram negative bacteria was determined using a kinetic-turbidimetric method (Talia et al., 2009, 2011, 2012). The strains were inoculated in 30 mL of a Müller-Hinton broth and incubated at 37°C for 20 h under gentle agitation. *H. pylori* strains were incubated in Müller Hinton broth supplemented with 5% fetal bovine serum and incubated at 37°C for 48 h under microaerophilic conditions.

The microbial growth kinetic assays were performed in 25 mL titration flasks with Müller-Hinton broth inoculated with a 200 μL of log-phase culture and increasing peptide concentrations. Titration flasks were placed in a GFL Shaking Incubator Orbital Motion (Model 3,031, Burgwedel, Germany) at 37°C, under stirring at 180 rpm. A control without peptide was carried out. Aliquots were removed every 30 min for 6 h and the transmittance (T) was read at λ: 720 nm. The transmittance (T) was related to Nt (Colony Forming Units (CFU)/mL) according to the following equations:


(3) $$\ln\;\mathrm{Nt}\;\left(Grampositive\right)=\;\;27.4\_10.3\;\;\times \;\;T\;$$

(4) $$\ln\;\mathrm{Nt}\;\left(Gramnegative\right)=\;\;27.1\_8.56\;\;\times \;\;T\;$$

Specific microbial growth rates were determined from ln Nt versus time plots.

The minimum inhibitory concentration (MIC) was obtained graphically, from the curve of the specific microbial growth rate versus the peptide concentration (μg/mL). MIC is established as the lowest concentration of a substance that inhibits the microbial growth after incubation in its presence (Andrews, 2001). The MIC of Nisin (a natural food preservative) and several synthetic antibiotics, frequently used in human and animal medicine, and often found in food, were used as control (Jensen et al., 2020; IDEXX Laboratories Inc©, 2022). Besides, AMX was used as a positive control in assay with *H. pylori* strains.

### Statistical analysis

Residual proteolytic activity, degree of conversion (αs) of acyl donor substrate into product, and peptide yield (η) were obtained from three independent trials which were done by duplicate, and data were reported as mean ± SD. The linear range of proteolytic activity reaction was previously determined for each assay. IBM® SPSS® Statistics V22.0 software was used for statistical analysis.

## Results

### Granulosain stability

Plant extracts, such as that from the fruits of *Solanum granuloso leprosum*, contain many water-soluble components, including pigments, phenolic compounds, and carbohydrates. These components usually interfere with the expression of enzymatic activity and therefore it is necessary to purify the proteins.

The use of organic solvents, such as acetone, allows to obtain a solid protein concentrate which can be dissolved in an appropriate buffer solution. This partially purified enzyme extract is jointly called granulosain and retained high enzyme activity (>90%; Vallés et al., 2008).

The residual proteolytic activity of granulosain (2.47 mg/mL, 32.49 ± 0.186 IU/mg) after 8 h in liquid – liquid aqueous organic systems formed by 30, 50, and 70% (v/v) of different immiscible organic solvents in 0.1 M Tris–HCl buffer pH 8 at 40°C, is shown in Table 1.

**Table 1:** Residual proteolytic activity of granulosain after 8 h in liquid–liquid biphasic media formed by different percentages of organic solvent in 0.1 M Tris HCl buffer pH 8, at 40°C.

Organic solvent	Residual proteolytic activity (IU/mg)		
	30%	50%	70%
Toluene	3.00 ± 0.00	4.13 ± 0.173	6.14 ± 0.161
1-Heptanol	0.33 ± 0.175	0.49 ± 0.141	0.00 ± 0.000
1,2-Dichloroethane	11.93 ± 0.205	29.05 ± 0.185	3.89 ± 0.210
Isopropyl ether	0.33 ± 0.144	0.41 ± 0.112	0.00 ± 0.000
n-Hexane	14.90 ± 0.186	23.71 ± 0.109	7.29 ± 0.124
2-Chlorotoluene	12.90 ± 0.06	27.21 ± 0.214	7.02 ± 0.227
Phenyl acetone	0.33 ± 0.175	0.41 ± 0.042	0.00 ± 0.000
Ethyl acetate	19.63 ± 0.210	31.98 ± 0.209	0.148 ± 0.134

Residual proteolytic activity of granulosain in 0.1 M Tris–HCl buffer pH 8 at 40°C was 32.49 ± 0.186 (IU/mg).

Granulosain exhibited similar or slightly lower residual proteolytic activity in several biphasic media, such as 50% (v/v) ethyl acetate, 1,2-dichloroethane, 2-chlorotoluene and n-hexane, than in 0.1 M Tris–HCl buffer pH 8. Those values ranged from 73 to 99% of the residual proteolytic activity obtained in buffer under the same conditions. Particularly, in 30% (v/v), 50% (v/v), and 70% (v/v) ethyl acetate in 0.1 M Tris–HCl buffer pH 8 at 40°C the following residual proteolytic activity values (IU/mg) were obtained: 19.63 ± 0.210, 31.98 ± 0.209, and 0.148 ± 0.134, respectively. In other biphasic media (toluene, 1-heptanol, isopropyl ether and phenyl acetone) granulosain showed little or no proteolytic activity. In addition, in all liquid–liquid biphasic media, when the organic solvent concentration was increased to 70% (v/v) the residual proteolytic activity of granulosain decreased sharply or was null under the studied conditions.

Some factors that contribute to the loss of enzymatic activity in organic media, such as the partitioning of immiscible organic solvents in the aqueous phase and the increase of the interfacial area, has been reported in the literature (Illanes et al., 2013; Kumar et al., 2016). Several cysteine proteases from South American plants of the family Asclepiadaceae, such as araujiain from the latex of fruits of *Araujia hortorum* Fourn., funastrain from the latex of stems of *Funastrum clausum* (Jacq.) Schlechter, and asclepain from *Asclepias curassavica* L.; expressed higher enzymatic activity in biphasic media than in buffer solution (Barberis et al., 2006; Quiroga et al., 2006, 2007; Origone et al., 2018). However, enzymes from the same family may show different behavior toward organic solvents. Cysteine proteases are hydrolase enzymes that share a common catalytic mechanism involving a nucleophilic cysteine thiol in a catalytic triad or diad. Organic solvents can act not only around the active site of the enzyme but also on the surface of the protein, modifying their interactions and leading to structural and functional changes in the protein. Such changes sometimes lead to more active enzyme structures or may cause loss of enzyme activity.

Ethyl acetate in 0.1 M Tris–HCl buffer pH 8 at 50% (v/v) was selected as the reaction medium for the synthesis of the Z-IQ peptide based on the high proteolytic activity and stability of granulosain, as also due to the adequate solubility of the substrates and their partition coefficients to the aqueous phase where the enzyme is dissolved.

### Granulosain preferences for synthetic amino acids

**Figure 1:**
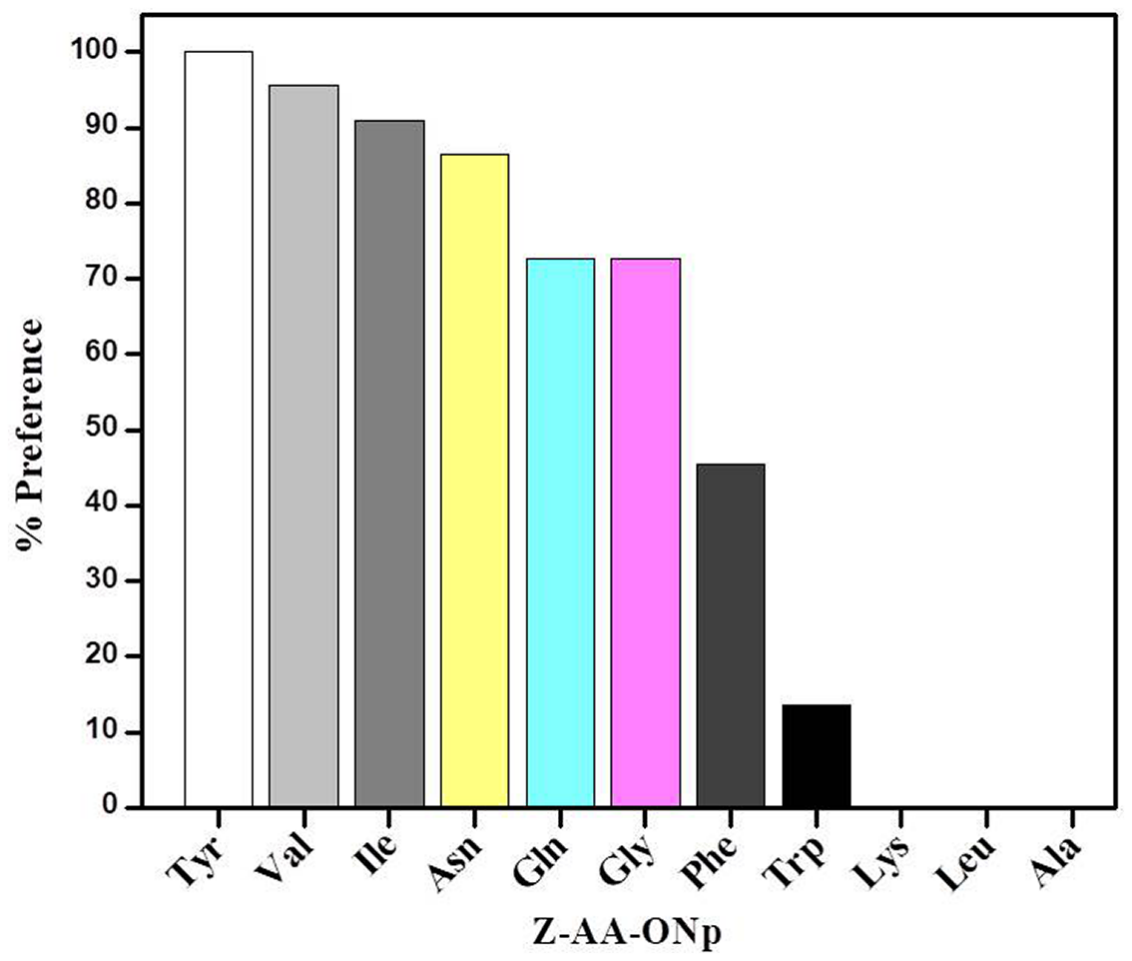
Preferences of granulosain for N-α-[Carbobenzyloxy]-amino acid-p-nitrophenyl esters (Z-AA-ONp) in 50% (v/v) ethyl acetate in 0.1 M Tris–HCl buffer pH 8, at 40°C.

Figure 1 shows the preferences of granulosain for N-α-[Carbobenzyloxy]-amino acid-p-nitrophenyl esters in 50% (v/v) ethyl acetate in 0.1 M Tris–HCl buffer pH 8, at 40°C. Granulosain in 50% (v/v) ethyl acetate in 0.1 M Tris–HCl buffer pH 8 showed high preferences for Ile, Val and Tyr derivatives.

In the enzymatic synthesis of peptides, amino acids acting as acyl donor and nucleophile are recognized by the S subsite and S′ region of the enzyme, respectively. The specificity of the enzyme for the acyl donor defines the rate of the reaction, while the specific binding of the nucleophile to the S′ subsite of the protease is necessary to achieve high yields (Schechter and Berger, 1967).

The high preference of granulosain for Z-I-ONp suggests that it would be a good acyl donor substrate and Gln OH was selected as nucleophile for the peptide synthesis reaction.

### Enzymatic synthesis of carboxyl-terminal Z-IQ peptide

The concentration of the acyl donor was established after determining the Z-I-ONp solubility in the organic phase of the reaction medium (50% (v/v) ethyl acetate in 0.1 M Tris–HCl buffer pH 8) at 40°C and 200 rpm, which was 0.318 mM; the partition coefficient of Z-I-ONp between the phases (P: 4.30), and the kinetic parameters of granulosain I (0.6158 mM; Vallés et al., 2008).

The partition coefficient of Z-I-ONp in the selected reaction medium was several times higher than in other biphasic media. Consequently, 50% (v/v) ethyl acetate in 0.1 M Tris–HCl buffer pH 8 at 40°C not only allowed to express high activity and stability to granulosain but also allowed a high concentration of acyl donor to be available in the aqueous phase where the enzyme is dissolved.

**Figure 2:**
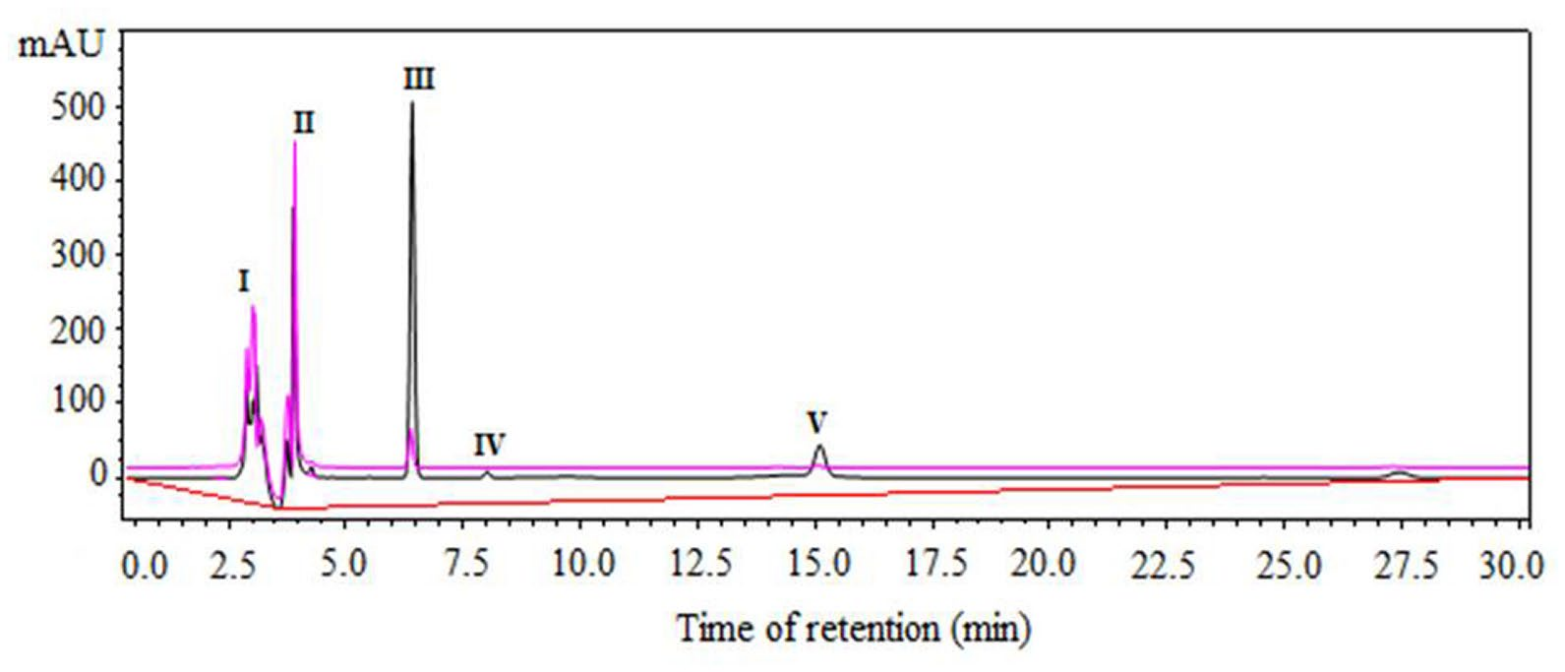
Component separation by RP-HPLC of a representative sample from N-α-CBZ-Ile-Gln-OH (Z-IQ) enzymatic synthesis under kinetic control, using granulosain (2.47 mg/mL, 32.49 ± 0.186 IU/mg) as soluble biocatalysts in 50% (v/v) ethyl acetate in 0.1 M Tris–HCl buffer pH 8, after 1 h of reaction at 40°C and 200 rpm. I: granulosain (t_R_: 2,7–3,2 min); II: N-α-CBZ-Ile-OH (Z-I-OH) (t_R_: 4 min); III: N-α-CBZ-Ile-Gln-OH (Z-IQ) (t_R_: 6.5 min); IV: 4-Nitrophenol (ONp) (t_R_: 8.0 min); V: N-α-CBZ-Ile-ONp (Z-I-ONp) (t_R_: 15.2 min). Aqueous phase: pink line. Organic phase: black line.

Figure 2 shows the separation of reactants and products by RP-HPLC from a representative sample of the enzymatic synthesis of Z-IQ under kinetic control, after 1 h of reaction. According to Figure 2, at a retention time (t_R_) of 6.5 min a peak of the main product (III) was observed in the organic phase. After 30 min, the main product was hydrolyzed to Z-I-OH (II) and partitioned to the aqueous phase. The Z-IQ peptide could be separated easily from the byproduct by stopping the agitation at the end of the reaction.

Table 2 shows the Z-IQ product yields (*η*) and the conversion percentage of acyl donor substrate into product (αs) for the enzymatic synthesis under kinetic control of Z-IQ peptide, using 50% (v/v) ethyl acetate in 0.1 M Tris–HCl buffer pH 8 as reaction medium and granulosain as biocatalysts, at 40°C and 200 rpm.

**Table 2:** Product yield (η) and conversion percentage of acyl donor substrate (αs) in the kinetically controlled synthesis of Z-IQ in 50% (v/v) ethyl acetate in 0.1 M Tris–HCl buffer pH 8, using granulosain as biocatalysts, at 40°C and 200 rpm.

Time (min)	Z-IQ (mM)	Z-I-OH (mM)	αs (%)	η (%)
0	0.000 ± 0.00	0.000 ± 0.00	0.0	0.0
1	0.027 ± 0.01	0.009 ± 0.00	9.0	6.9
5	0.248 ± 0.03	0.027 ± 0.01	70.1	63.3
15	0.278 ± 0.00	0.063 ± 0.01	87.3	**71.0**
30	0.252 ± 0.02	0.131 ± 0.01	97.7	64.3
60	0.239 ± 0.01	0.153 ± 0.00	100	61.0
180	0.175 ± 0.00	0.217 ± 0.00	100	44.6

The synthesis of Z-IQ under kinetically controlled conditions, and with one of the substrates in excess, allowed to increase the selective conversion toward a single product until 71% after 15 min, remaining unconverted 12.7% of the limiting substrate remaining unconverted. These results revealed the high specificity and catalytic capacity of granulosain.

Peptide synthesis reactions under kinetic control in aqueous-organic media proceed via an acyl-enzyme intermediate, which reacts with the N-terminal nucleophile and becomes the C-terminal segment of the peptide product. However, the nucleophile competes with water for cleaving that intermediate and to form either a peptide or a hydrolyzed substrate. In fact, the hydrolysis of the acyl donor substrate decreased the product yield in the synthesis reaction of Z-IQ. When the thermodynamic equilibrium conditions prevailed the reaction did not proceed.

The reaction product was separated from the organic phase, purified by means of a C_18_ column, dried with a concentrator equipment (Thermo Scientific SavantTM SPD 131 DDA Speed Vac) and analyzed by electrospray ionization mass spectrometry (LCMS-2020, Shimazu).

**Figure 3:**
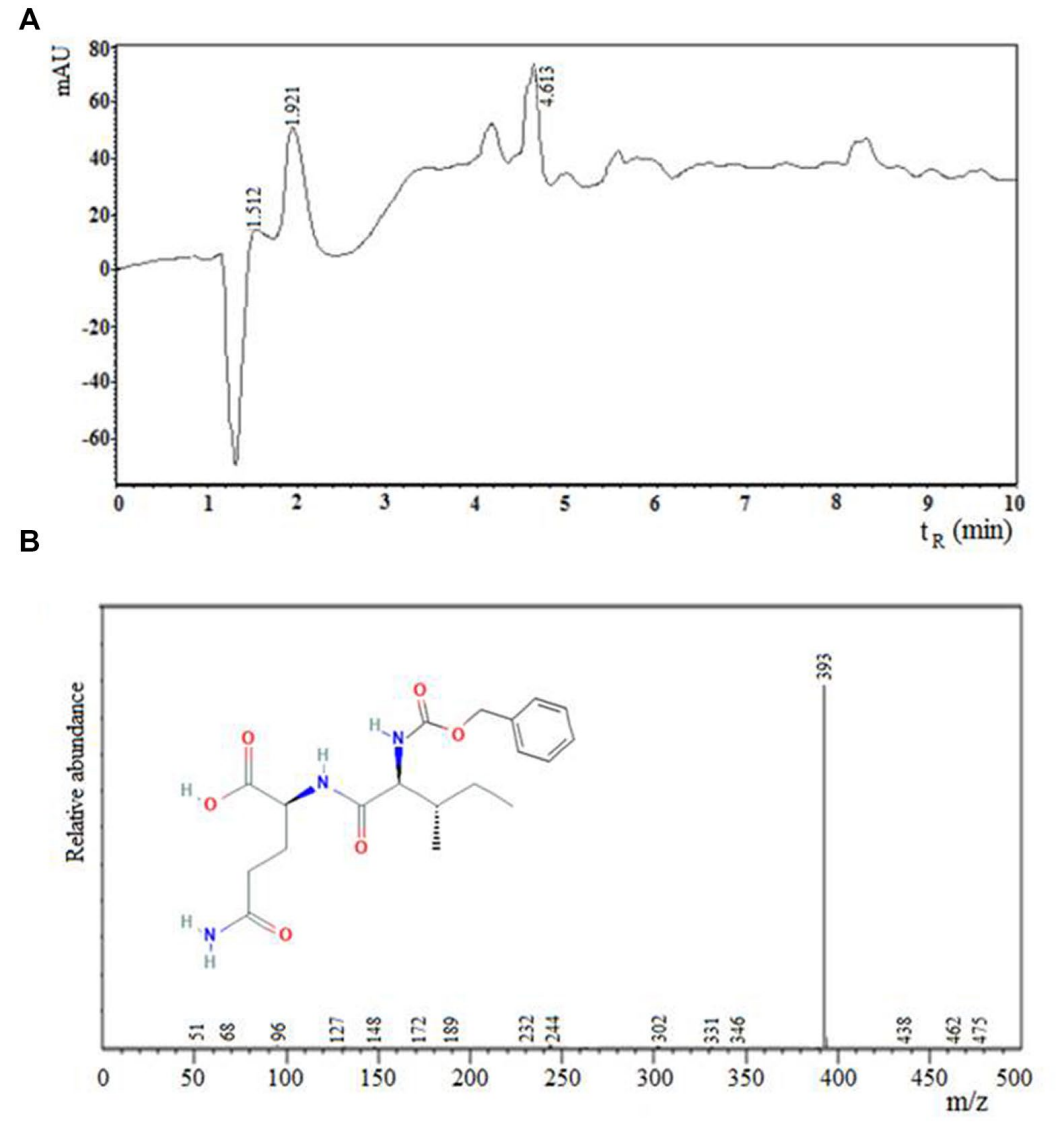
Chromatogram obtained by **(A)** RP-HPLC and **(B)** mass spectrum of the carboxyl terminal peptide N-α-CBZ-Ile-Gln-OH (Z-IQ). The mass spectrum of the main product of the enzymatic synthesis reaction (III, t_R_: 6.5 min) showed ion mass (m/z): 393, corresponding to the peptide Z-IQ.

Figure 3 shows the chromatogram obtained by (a) RP-HPLC and (b) mass spectrum of the carboxy terminal peptide N-α-CBZ-Ile-Gln-OH (Z-IQ). The mass spectrum of the main product of the enzymatic synthesis reaction (III, t_R_: 6.5 min) showed ion mass (m/z): 393, corresponding to the peptide Z-IQ.

### Chemical synthesis of amine-terminal IQ peptide

**Figure 4:**
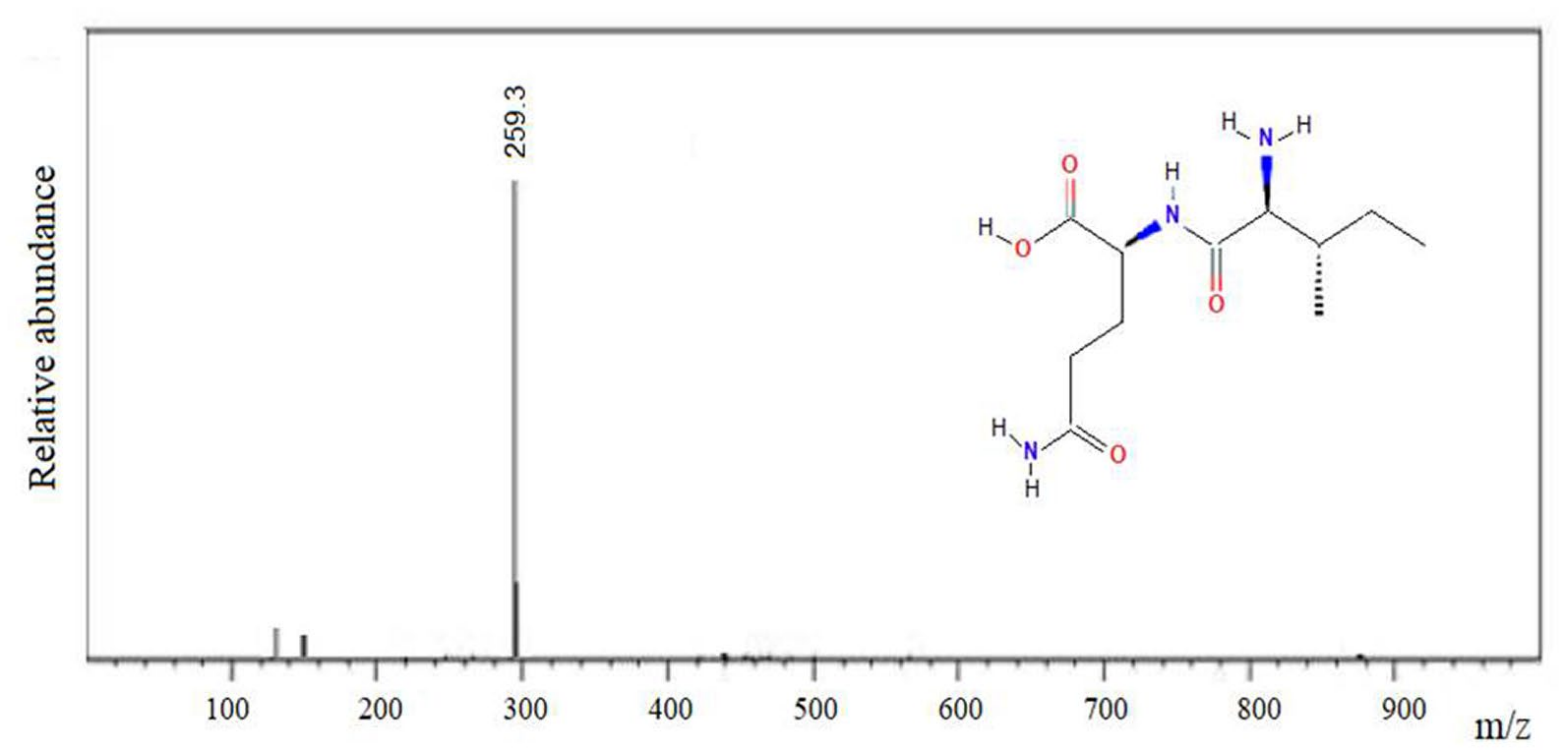
Mass spectrum of the amine-terminal IQ peptide, m/z: 259.3.

The IQ peptide was chemically synthesized using Nα-Fmoc strategy, as it was previously described. The purity was higher than 95% and the molecular mass was confirmed by MALDI-TOF mass spectrometry (Figure 4).

### Antibacterial activity of Ile-Gln (IQ)

Antibacterial activity of Ile-Gln (IQ) was tested through three independent trials, in duplicate, against Gram positive and Gram negative strains in batch culture with Müller-Hinton broth at 37°C under agitation at 180 rpm, using increasing concentrations of IQ (0–500 μg/mL).

Table 3 shows the minimum inhibitory concentrations (MIC) values of IQ against three reference strains: *S. aureus* ATCC 25923, *E. coli* ATCC 25922, and *H. pylori* NCTC 11638, and six Gram positive and Gram negative type-wild strains isolated in the Microbiology Laboratory of the Regional Polyclinic of San Luis, Argentina, and three wild-type *H. pylori* strains isolated in our laboratories from biopsy samples of the gastric antrum of patients from San Luis, Argentina.

**Table 3:** Minimum inhibitory concentrations (MIC) values of Ile-Gln-OH dipeptide against Gram positive and Gram negative strains.

Microorganism		MIC				
	Ile-Gln-OH (μg/mL)	1	2	3	4	5
			(μg/mL)			
*Gram positive bacteria*						
*Staphylococcus aureus* ATCC 25923	133.7 ± 0.05	8–16	6.4–12.8	0.4	2–4	8–32
*Staphylococcus aureus* C00195	118.0 ± 0.01					
*Staphylococcus hominis* A17771	125.0 ± 0.05					
*Enterococcus faecalis* I00125 (MDR)	–					
*Gram negative bacteria*						
*Escherichia coli* ATCC 25922	82.4 ± 0.01	8–32	––	0.4	8–32	4–16
*Escherichia coli* A17683	85.0 ± 0.00					
*Klebsiella oxytoca* A19438 (SDR)	–					
*Pseudomonas aeruginosa* C00213 (SDR)	–					
*Helicobacter pylori* NCTC 11638^1^	250 ± 0.01					
*Helicobacter pylori* 659^1^	500 ± 0.01					
*Helicobacter pylori* 661 (SDR)^2^	500 ± 0.01					
*Helicobacter pylori* 662 (SDR)^3^	500 ± 0.01					

The MIC of Nisin (a natural food preservative) and several synthetic antibiotics often found in food, were used as control: **1.** Ampicillin, **2.** Nisin, **3.** Erythromycin, **4.** Chloramphenicol, **5.** Gentamicin.

1 Sensitive to AML, MTZ, LEV, and CLA. Pathology: Chronic gastritis.

2 Resistant to LEV. Pathology: Gastric ulcer.

3 Resistant to MTZ. Pathology: Chronic gastritis.

The new dipeptide (IQ) caused growth inhibition of Gram-positive bacteria, such as *S. aureus* ATCC 25923, *S. hominis* A17771 and *S. aureus* C00195, between 118 ± 0.01 μg/mL and 133.7 ± 0.05 μg/mL; but did not inhibit the growth of the multidrug resistant *E. faecalis* I00125 (MDR). In addition, IQ showed MIC of 82.4 ± 0.01 μg/mL and 85.0 ± 0.00 μg/mL against *E. coli* ATCC 25922 and *E. coli* A17683, respectively, but did not show inhibitory activity against simple resistant strains, such as *Klebsiella oxytoca* A19438 (SDR) and *Pseudomonas aeruginosa* C00213 (SDR).

IQ also inhibited the microbial growth of *H. pylori* NCTC 11638 and three wild-type *H. pylori* strains isolated from antral-gastric biopsies of patients from San Luis, Argentina. MIC values of 250 ± 0.01 μg/mL and 500 ± 0.01 μg/mL were obtained for *H. pylori* NCTC 11638 and the other *H. pylori* strains, respectively. MIC of synthetic antibiotics frequently used in human and animal medicine; and often found in food, were used as control (IDEXX Laboratories Inc©, 2022).

**Figure 5:**
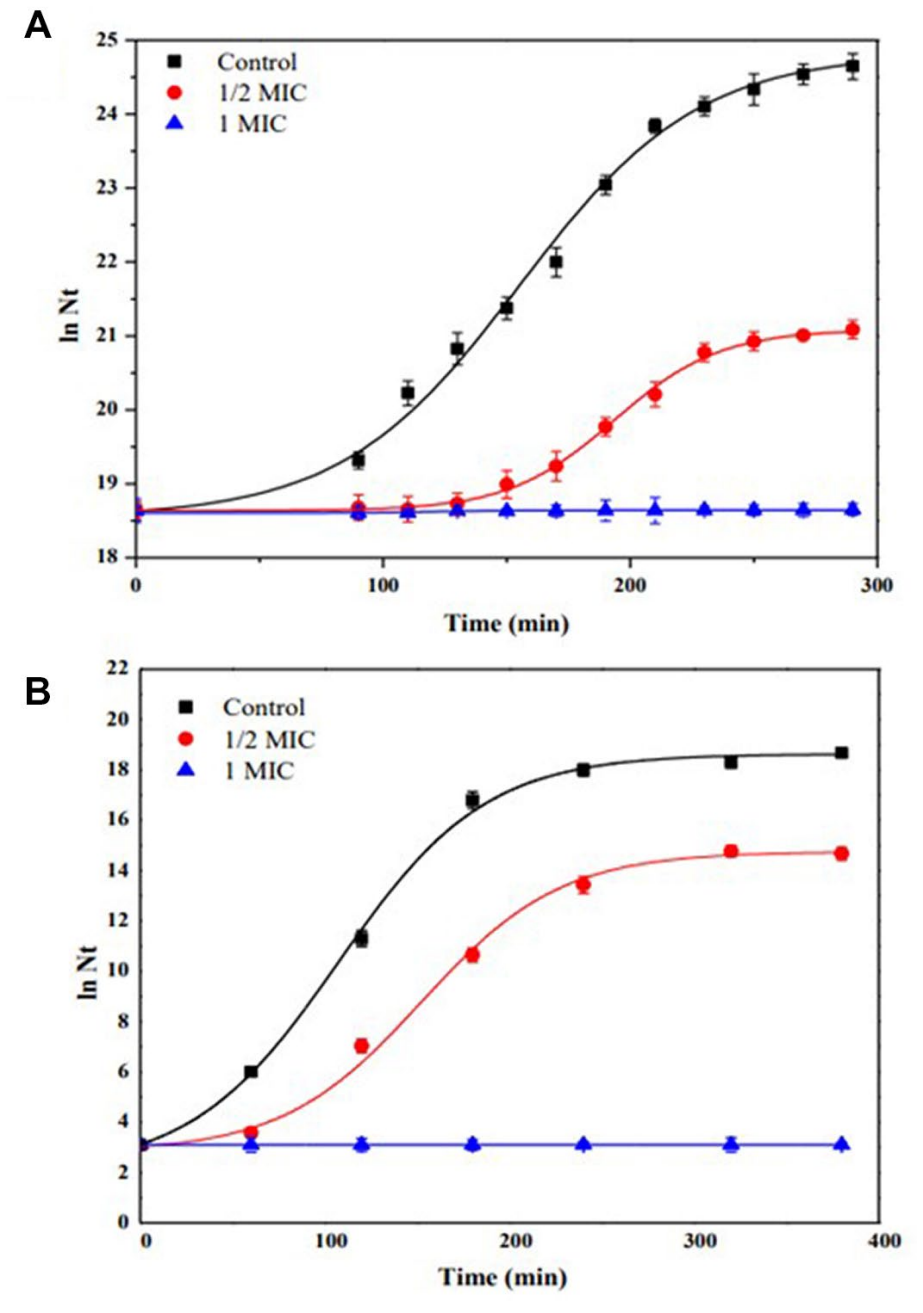
Growth kinetics in batch culture of **(A)** *Staphylococcus aureus* C00195, using Müller-Hinton broth and increasing concentration of IQ (0 to 118 μg/mL) at 37°C and 180 rpm. **(B)** *Helicobacter pylori* NCTC 11638, using Müller-Hinton broth supplemented with 5% fetal bovine serum, and increasing concentration of IQ (0 to 250 μg/mL), at 37°C under microaerophilic conditions. Control (without IQ).

Figure 5 shows the kinetics of growth in batch culture of (A) *S. aureus* C00195, using Müller-Hinton broth and increasing concentration of IQ (0 to 118 μg/mL) at 37°C and 180 rpm, (B) *H. pylori* NCTC 11638, using Müller-Hinton broth supplemented with 5% fetal bovine serum, and increasing concentration of IQ (0 to 250 μg/mL), at 37°C under microaerophilic conditions. Control (without IQ).

**Figure 6:**
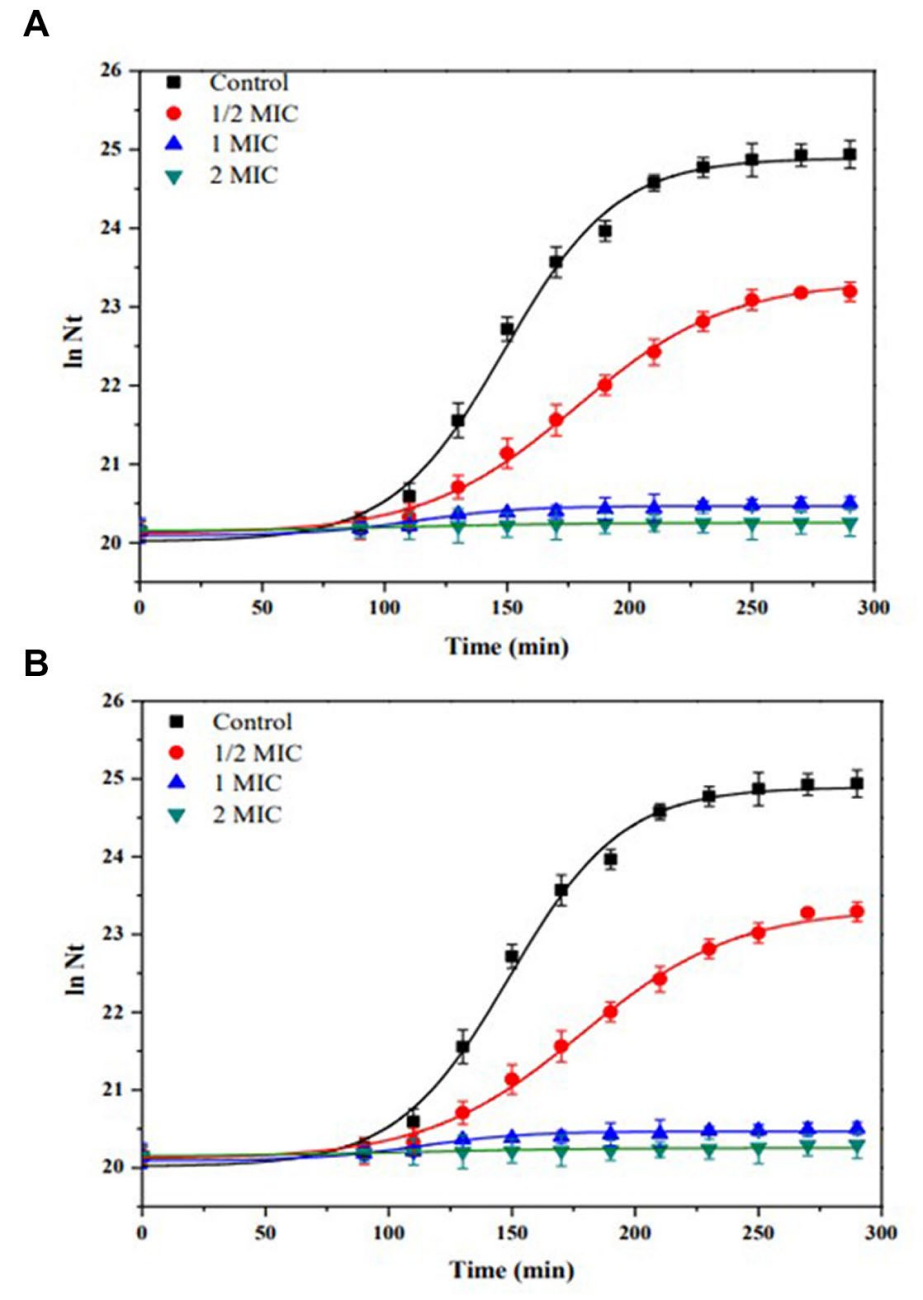
Growth kinetics in batch culture of *Escherichia coli* ATCC 25922, using Müller-Hinton broth and increasing concentration of **(A)** IQ (0 to 164.8 μg/mL). **(B)** Z- IQ (0 to 164.8 μg/mL), at 37°C and 180 rpm. Control (without IQ).

Figure 6 shows the kinetics of growth in batch culture of *E. coli* ATCC 25922, using Müller-Hinton broth and increasing concentration of (A) IQ (0 to 164.8 μg/mL), (B) Z- IQ (0 to 164.8 μg/mL), at 37°C and 180 rpm.

As shown in Figure 6, benzyloxycarbonyl group (Z) did not significantly modify the bacterial growth inhibitory activity of the IQ dipeptide. In contrast, the carboxyl terminus of the IQ dipeptide was essential for antibacterial activity. In fact, the amine-terminal dipeptide IQ-NH_2_ had no antibacterial activity against the strains studied.

Those results suggest that the new dipeptide (IQ-OH) can be used as an antimicrobial agent against sensitive Gram positive and Gram negative bacteria, for food preservation or as an ingredient of functional foods.

It is important to highlight that the IQ-OH sequence has not yet been reported in bioactive peptide databases (Dziuba et al., 1999; Shtatland et al., 2007; Liu et al., 2008; Thomas et al., 2010; Wang et al., 2017), and according to our results, it is a promising antimicrobial agent.

## Discussion

Synthetic antibiotics are known to be more effective than natural antimicrobial agents, such as AMPs. However, AMPs do not produce resistance and are safe at higher concentrations (Adaro et al., 2021). Besides, as food preservatives, it is expected that small peptides (such as IQ) can be rapidly hydrolyzed in the digestive system.

Peptide therapies are currently targeted at the treatment of cancer (goserelin, bortezomib, leuprorelin), cardiovascular (bivalirin, eptifibatide) and central nervous system (glatiramer) diseases, infections (telaprevir, boceprevir), metabolic disorders (liraglutide, exenatide), hematological (icatibant, ecallantide) and gastrointestinal (teduglutide, linaclotide) diseases, among others (Yu and Rao, 2014; Fosgerau and Hoffmann, 2015; Zhang et al., 2016).

In contrast to the widespread development of therapeutic peptides, the commercial production of bioactive peptides with specific properties for food use (nutraceuticals, functional foods, and preservatives) is rare, although it is an area of intense research. Nisin (of bacterial origin) and lysozyme (of animal origin), designated E234 and E1105 in the JECFA list of food additives, are so far the only peptides approved for marketing by the WHO (World Health Organization, 2010).

Soluble granulosain catalyzed the Z-IQ dipeptide synthesis under kinetic-control with high yield (71 ± 0.10%) using an aqueous–organic biphasic system formed by 50% (v/v) ethyl acetate and 0.1 M Tris – HCl buffer pH 8. Nowadays, the molecular effect of organic solvents on the activity, operational stability, flexibility and secondary structure of granulosain, using different aqueous-organic biphasic media, are being evaluated.

This paper reports a novel carboxyl-terminal antibacterial peptide (Ile-Gln-OH) that significantly decreased (*p* ≤ 0.05) the specific growth rates of several Gram positive and Gram negative sensitive strains, at low concentrations. Particularly, MIC values from 118 ± 0.01 μg/mL to 133.7 ± 0.05 μg/mL for several *S. aureus* strains and from 82.4 ± 0.01 μg/mL to 85.0 ± 0.00 μg/mL for *E. coli* strains were obtained. These MIC values were several time lower than others reported in the literature for *S. aureus* and *E. coli* strains, using novel dipeptides (Soares et al., 2023).

IQ also inhibited the microbial growth of *H. pylori* NCTC 11638 at ≥250 ± 0.01 μg/mL and three wild-type *H. pylori* strains at ≥500 ± 0.01 μg/mL. It is important to note that, among these last three strains, one is sensitive to AML, MTZ, LEV and CLA (*H. pylori* 659), another is resistant to LEV (*H. pylori* 661), and the third is resistant to MTZ (*H. pylori* 662).

Finally, this study contributes with a new dipeptide (IQ) that can be used as an antimicrobial agent for food preservation or as a safe ingredient of functional foods.

Further studies will be conducted to elucidate the mechanism of action of IQ against those bacterial strains.

## Data availability statement

The original contributions presented in the study are included in the article/Supplementary material, further inquiries can be directed to the corresponding author.

## Ethics statement

Ethical review and approval was not required for this study in the Universidad Nacional de San Luis, San Luis, Argentina, because the samples were collected during routine care and the patients fulfilled the written informed consent form at Healthcare Centers of San Luis, Province of San Luis, Argentina, prior to sample collection. The isolated strains were later donated to the Faculty of Chemical, Biochemical and Pharmacy of the Universidad Nacional de San Luis.

## Author contributions

SB designed the experiments, did the data analyzing, and manuscript writing. MA did the experimental assays, data collection, and analysis. AI, AV, and AO collaborated in the antimicrobial activity experiments. DV collaborated in the preparation of partially purified enzymatic extracts. FG collaborated in the chemical synthesis and analysis of the IQ-NH_2_ peptide. All authors contributed to the article and approved the submitted version.

## Funding

This research was funded by the National University of San Luis, San Luis, Argentina (Grant number 2–0718, 2018–2022). MA and AI are Postdoctoral Fellows at CONICET, Argentina. SB is Researcher Career Member at CONICET, Argentina.

## Conflict of interest

The authors declare that the research was conducted in the absence of any commercial or financial relationships that could be construed as a potential conflict of interest.

## Publisher’s note

All claims expressed in this article are solely those of the authors and do not necessarily represent those of their affiliated organizations, or those of the publisher, the editors and the reviewers. Any product that may be evaluated in this article, or claim that may be made by its manufacturer, is not guaranteed or endorsed by the publisher.
