# Supplementary figures and images for: Enzymatic synthesis of new antimicrobial peptides for food purposes
Source: Front Microbiol. 2023 May 16;14:1153135. doi: 10.3389/fmicb.2023.1153135 (PMC10227576; doi:10.3389/fmicb.2023.1153135)

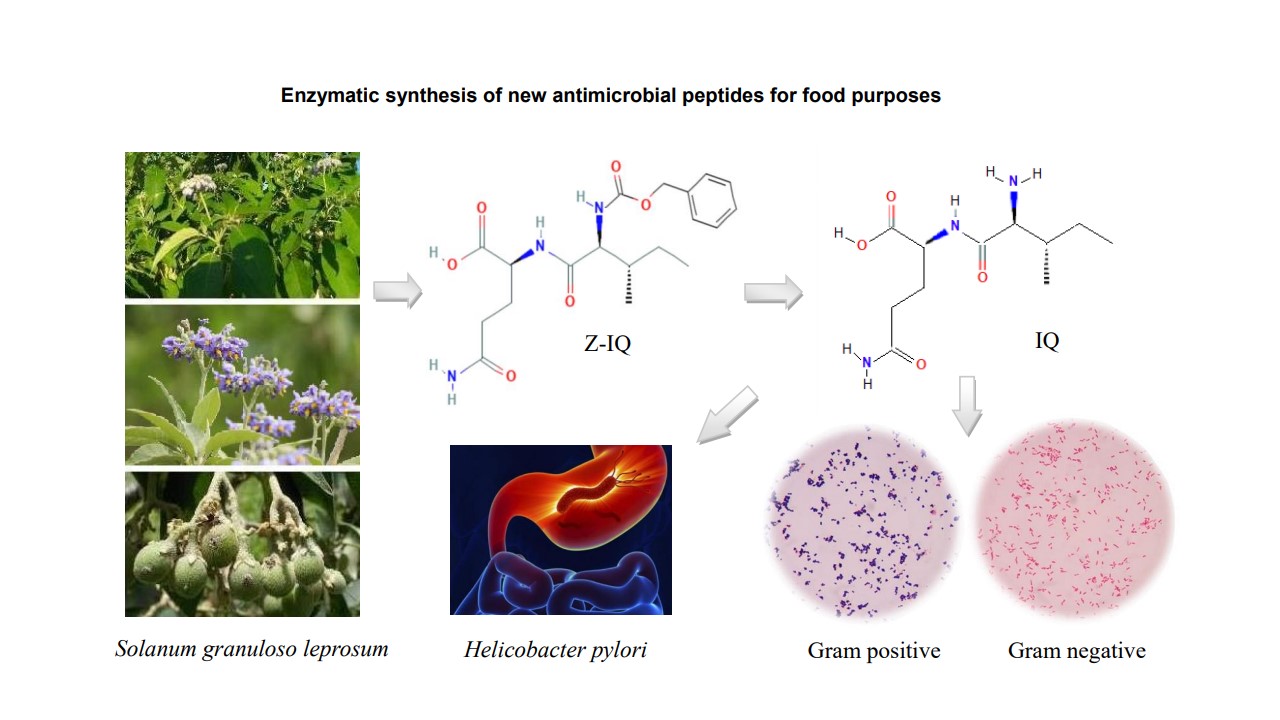

Supplement: Supplementary file 1 [file Image_1.JPEG]
